# Uneven development of intangible cultural heritage: A spatial analysis of traditional drama in Southwestern China

**DOI:** 10.1371/journal.pone.0355102

**Published:** 2026-08-14

**Authors:** Yun Qin, Zhiyi Zeng, Keyao Qin, Jiexi Xiong, Xiang Yan

**Affiliations:** 1 Academy of Fine Arts and Design, Chengdu Normal University, Chengdu, China; 2 School of Tourism, Northwest Normal University, Lanzhou, China; 3 Institute of Urbanization Strategy and Architecture Research, Sichuan University, Chengdu, China; Rikkyo University: Rikkyo Daigaku, JAPAN

## Abstract

Intangible cultural heritage (ICH) exhibits pronounced spatial unevenness, yet the spatial patterns and driving mechanisms of traditional drama as a form of living performing heritage remain insufficiently understood. Taking Southwest China as a case, this study analyzes 257 traditional drama ICH items, comprising 54 national-level and 203 provincial-level items. Using kernel density estimation, nearest neighbor index, standard deviational ellipse, and the geographical detector methods, we examine spatial distribution, typological differentiation, temporal evolution, and influencing factors. The results show that: (1) traditional drama ICH displays a “dense in eastern section while sparse in western section” pattern, with core clusters in eastern Guizhou and the Sichuan–Chongqing–Guizhou border region; (2) clear typological differences exist, with Dengxi Opera, Tibetan Opera, and small-scale dramas (Others) showing clustered patterns, while Sichuan Opera, Di Opera, and shadow puppetry are more dispersed; (3) from 2006 to 2025, the spatial pattern evolves from scattered distribution to multi-core agglomeration, with a relatively stable gravity center in the Sichuan–Chongqing–Guizhou border area; and (4) terrain, traditional villages, and cultural institutions are the main driving factors, with interaction effects enhancing explanatory power, while economic and urbanization variables show no significant independent effects. This study responds to critiques of spatial disconnection from ICH’s living attributes and integrates cultural ecology, therapeutic arts, and digital heritage perspectives. It provides empirical support for the spatial governance and living preservation of performing ICH.

## 1. Introduction

Intangible Cultural Heritage (ICH) constitutes an important carrier of historical memory, ethnic culture and regional civilization, and its spatial distribution pattern has gradually become a key topic in the fields of cultural geography and heritage protection [1–5]. As a typical performing art category of ICH, traditional drama is rooted in local folk customs and social life, and its formation and inheritance are closely related to economic development, regional natural environment, and cultural factors [6, 7]. In recent years, with the wide application of GIS spatial analysis methods, scholars have carried out abundant studies on the spatial distribution of ICH at national, regional and provincial scales [8–11]. However, most existing studies focus on the overall ICH or single craft heritage, and there is still a lack of systematic and in-depth investigations on the spatial differentiation and driving mechanism of traditional drama at the regional scale [12–14].

Internationally, research on heritage space has increasingly moved beyond static inventories toward relational interpretations of cultural landscape, community practice, and spatial governance. European studies, for example, have examined cultural heritage as a tool for territorial cohesion, planning integration, and landscape resilience [6,9,10], while studies of urbanization and heritage in Vietnam and Tehran have shown how modernization reshapes the spatial conditions of cultural continuity [17,18]. For performing arts, research on live music spaces further emphasizes that performance does not simply occupy a fixed location, but produces spatial value through repeated practice, community participation, and narration of place [28]^.^ These international discussions provide an important comparative lens for understanding traditional drama ICH as a living performing heritage embedded in regional cultural ecologies rather than as a set of isolated points.

Southwest China, including Sichuan, Chongqing, Guizhou, Yunnan and Xizang, is characterized by complex and diverse landforms, large altitude differences, numerous ethnic groups and rich cultural traditions. This region has nurtured a large number of traditional drama types with distinctive regional and ethnic features, such as Sichuan Opera, Tibetan Opera, Dengxi Opera and Nuo Opera, providing a typical case for understanding the spatial distribution law of performing ICH. At present, studies on traditional drama in Southwest China mostly focus on artistic value, inheritance dilemma and protection strategies, while few studies reveal its spatial agglomeration characteristics, provincial differences, temporal evolution and influencing factors from a holistic regional perspective [15].

Therefore, this study takes traditional drama ICH in Southwest China as the research object, and takes 257 national-level and provincial-level items as data samples. By using kernel density estimation, nearest neighbor index, standard deviational ellipse and geographical detector model, this paper systematically analyzes the spatial distribution pattern, type differentiation, temporal evolution characteristics and driving mechanism of traditional drama ICH in Southwest China. The purposes of this study are: first, to clarify the overall spatial pattern and provincial differences of traditional drama ICH in Southwest China; second, to reveal the agglomeration and dispersion characteristics of different drama types; third, to identify the key factors affecting the spatial distribution of traditional drama ICH.

This study focuses on the regional scale of Southwest China, and expands the empirical research on the spatial distribution of performing ICH. The results can not only deepen the understanding of the relationship between traditional drama and regional environment, but also provide a scientific reference for the spatial governance, coordinated protection and targeted inheritance of traditional drama ICH in Southwest China.

From this perspective, Southwest China is used not only as a local Chinese case, but also as a regional case through which broader questions in international ICH research can be examined: how living performing heritage is spatially anchored without being reduced to immobile sites; how cultural ecology, settlements, and public cultural institutions shape inheritance conditions; and how uneven regional development affects heritage safeguarding. The findings may therefore offer comparative implications for other multicultural and mountainous regions where performing traditions are transmitted through communities, festivals, itinerant practice, and local cultural spaces.

## 2. Materials and methods

### 2.1 Study area

**Fig 1:**
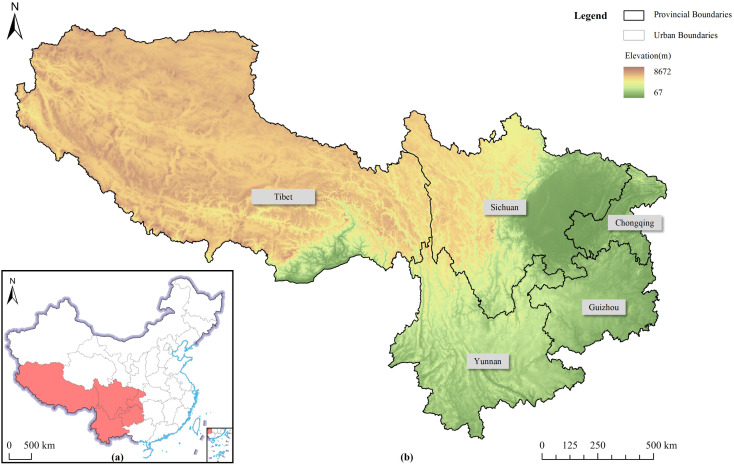
Study area (standard map from the China National Geographic Information Public Service Platform website, with the approval number GS (2023) 2767).

This study selects traditional drama from five provinces/autonomous regions in Southwest China as the research object to analyze their spatial distribution characteristics. Southwest China is an important area rich in cultural resources in China, covering five provincial-level administrative regions: Sichuan, Chongqing, Yunnan, Guizhou, and the Tibet Autonomous Region (Fig 1). The geographical environment is complex and diverse, including geomorphological units such as the Sichuan Basin, the Yunnan-Guizhou Plateau, and the Qinghai-Tibet Plateau. This region is a typical area for the coexistence of diverse ethnic cultures in China. Due to its special historical background and geographical relations, it constitutes a regional culture with continuous lineage and has nurtured a rich variety of traditional drama ICH, such as Sichuan Opera, Yunnan Opera, Dong Opera, Tibetan Opera, etc. These traditional drama ICH are not only an important component of the ethnic culture in Southwest China but also a vivid manifestation of regional cultural diversity.

### 2.2 Data

The information and data of national-level intangible cultural heritage cited in this article are all sourced from the China Intangible Cultural Heritage Network (https://www.ihchina.cn), while the information and data of provincial-level intangible cultural heritage come from the annual publicized intangible cultural heritage protection lists of each province (as of 2025). The full list is provided in the Appendix.

There are a total of 257 traditional drama ICH items in Southwest China (including 54 national-level and 203 provincial-level items). Using the coordinate picking function of Baidu Maps, the geographical locations of traditional drama intangible cultural heritage in Southwest China were determined based on each protection unit. It should be noted that these coordinates represent the officially recorded protection units or core inheritance areas, rather than fixed performance sites of traditional drama.

The base map was made based on the standard map shared by the China National Geographic Information Public Service Platform website, with the approval number GS (2023) 2767; the DEM data of terrain elevation and other geographic spatial data came from the Geospatial Data Cloud (http://www.gscloud.cn/); other socio-economic data were obtained from the “China Urban Statistical Yearbook” (2025) and the statistical yearbooks of the 5 provincial administrative regions in Southwest China, and missing data were supplemented using statistical bulletins, etc. Using ArcGIS 10.6 and Excel, the coordinate data were processed to create a spatial distribution map of traditional drama in Southwest China.

### 2.3 Methods

(1) ***Kernel Density Estimation (KDE)***

Kernel density analysis is a statistical method used to analyze the distribution density of spatial point data, widely applied in fields such as geography, ecology, urban and rural planning, and cultural heritage research. It is a non-parametric estimation method used to derive a continuous density distribution from discrete spatial point data. It assigns a “kernel function” to each spatial point and estimates the density distribution across the entire study area through the superposition of kernel functions, effectively analyzing the agglomeration distribution of various elements in space [14]. The calculation formula is:


$$\begin{array}{c} f_{n}(x)=\frac{\text{1}}{nh_{n}}\sum_{j=\text{1}}^{n}k\left(\frac{x-x_{j}}{h_{n}}\right) \end{array}$$ (1)

Where: $f_{n}(x)$is the kernel density estimate at location. $k\left(\frac{x-x_{j}}{h_{n}}\right)$is the kernel function; *n* is the number of data points; and $x-x_{j}$ is the distance from the estimation point x to the ICH point $x_{j}$.

(2) ***Nearest Neighbor Index***

This index is primarily used to evaluate the distribution pattern of point elements in geographic space, determining whether they exhibit clustered, random, or uniform distribution. Its core idea is to quantify the spatial distribution characteristics of point elements by comparing the actual observed average nearest neighbor distance with the theoretical random distribution distance [2,16]. The calculation formula is:


$$\begin{array}{c} R=\frac{\text{1}}{\text{2}\sqrt{\frac{n}{A}}}=\frac{\text{1}}{\text{2}\sqrt{D}} \end{array}$$ (2)

Where: *R* is the nearest neighbor index; $\overset{―}{r_{I}}$ is the actual mean nearest neighbor distance; $\overset{―}{r_{E}}$ is the expected mean nearest neighbor distance under random distribution; When *R* = 1, it indicates that the traditional drama ICH is randomly distributed in space; *R* > 1 indicates uniform distribution; *R* < 1 indicates clustered distribution.

(3) ***Standard Deviation Ellipse***

From a global spatial perspective, based on the spatial characteristics of the research object, this method analyzes the central tendency, degree of dispersion, and directional trend of the geographic elements’ distribution, ultimately revealing their spatial distribution and spatiotemporal evolution process [11,16]. The calculation formulas are:


$$\begin{array}{c} SDE_{x}=\sqrt{\frac{\sum_{i=\text{1}}^{n}(x_{i}-\overset{―}{X}{)}^{\text{2}}}{n}} \end{array}$$ (3)

$$\begin{array}{c} SDE_{y}=\sqrt{\frac{\sum_{i=\text{1}}^{n}(y_{i}-\overset{―}{Y}{)}^{\text{2}}}{n}} \end{array}$$ (4)

Where: $x_{i}$ and $y_{i}$ represent the coordinates of the traditional drama ICH in Southwest China; $\left\{\overset{―}{X},\overset{―}{Y}\right\}$ represent the mean center of the traditional drama ICH; *n* is the total number of heritage items.

(4) ***Geographical Detector***

Geographical detector is a statistical tool for spatial analysis. It can detect the formation relationships of the spatial distribution of traditional drama ICH in Southwest China and test the spatial coupling between two variables [14,16].


$$\begin{array}{c} q=\left(N\sigma^{\text{2}}-\sum_{h=\text{1}}^{L}N_{h}\sigma_{h}^{\text{2}}\right)/N\sigma^{\text{2}} \end{array}$$ (5)

Where: N and $\sigma^{\text{2}}$ are the sample size and variance of the traditional drama ICH distribution; $N_{h}$ and $\sigma_{h}^{\text{2}}$ are the sample size and variance of the *h* stratum of the influencing factor; *L* is the number of strata for the influencing factor. The *q* value ranges between 0 and 1. A larger *q* value indicates that this factor has a greater influence on the spatial distribution of the traditional drama ICH.

## 3. Spatial distribution patterns

### 3.1 Kernel density

**Fig 2:**
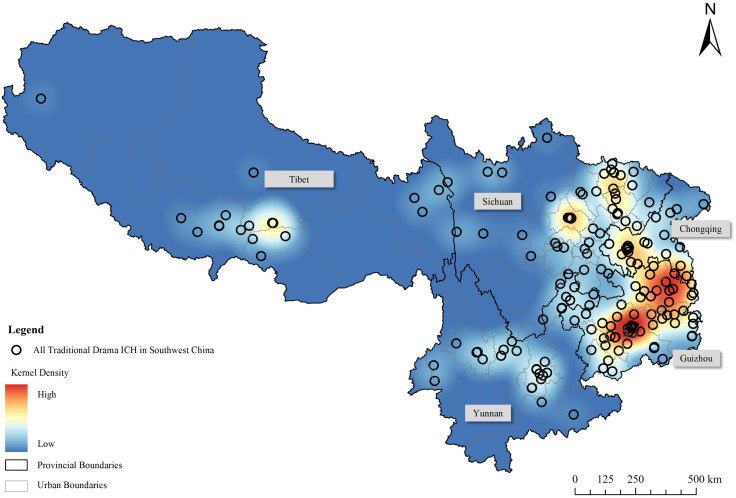
Kernel density of traditional drama ICH in Southwest China.

By using the kernel density analysis tool in ArcGIS to generate the kernel density map of traditional drama intangible cultural heritage (ICH) in Southwest China, the spatial distribution structure of these heritage items can be visually reflected (Fig 2). As shown in the figure, the traditional drama ICH in Southwest China presents an overall significant agglomeration distribution, with a distinct spatial pattern characterized by “dense in the east and sparse in the west, multi-core agglomeration, and gradient decline”, featuring extremely uneven distribution that is highly coupled with the quantity distribution. In terms of spatial structure, a gradient distribution structure is formed, with two core high-density agglomeration areas at the Sichuan-Chongqing-Guizhou border and central-eastern Guizhou, secondary agglomeration areas in northeastern Sichuan Basin and central Yunnan, and scattered distribution areas in plateau mountainous regions such as Xizang, western Sichuan, and western Yunnan. Among them, the Sichuan-Chongqing-Guizhou border forms a cross-provincial continuous high-value cultural agglomeration belt, while central-eastern Guizhou, centered on Guiyang, forms an independent high-value core area, serving as the core bearing highland of traditional drama ICH in Southwest China. From the quantitative perspective, the number of ICH projects in Guizhou, Chongqing, and eastern Sichuan accounts for the absolute majority of the total in Southwest China; Yunnan shows a point-like agglomeration centered on central Yunnan, while Xizang, western Sichuan, and western Yunnan in high-altitude areas have a small number of ICH projects with only sporadic distribution. Overall, a significant rule is presented that high agglomeration occurs in low-altitude densely populated areas and sparse distribution in high-altitude sparsely populated areas. This characteristic not only reflects the fundamental supporting role of natural and human conditions such as terrain and population in the survival of ICH, but also demonstrates the core driving effect of the Sichuan-Chongqing-Guizhou cultural integration area on the inheritance and development of traditional drama ICH.

### 3.2 Type and provincial differentiation characteristics

The National List of Intangible Cultural Heritage (ICH) of China classifies ICH into ten categories: folk literature, traditional music, traditional dance, traditional drama, quyi, traditional sports, entertainment and acrobatics, traditional fine arts, traditional craftsmanship, traditional medicine, and folk customs [16]. Since the release of the first batch in 2006, five batches of national-level representative ICH lists have been issued by December 2025, with a total of 3,661 sub-items, including 474 traditional drama ICH items. Among them, 54 national-level traditional drama ICH items are distributed in Southwest China, accounting for 11% of the national total, covering 24 opera types. In addition, the five provincial-level administrative regions have released 5–7 batches of provincial-level ICH lists (6 batches in Chongqing, 7 batches in Sichuan, 6 batches in Guizhou, 5 batches in Yunnan, and 6 batches in Xizang), with 203 confirmed provincial-level traditional drama ICH items. To further clarify the types and quantities of traditional drama ICH in each provincial-level region of Southwest China (Table 1).

**Table 1:** Types and geographical quantity distribution of traditional drama ICH in Southwest China.

No.	Type	Chongqing	Sichuan	Guizhou	Yunnan	Tibet	Total
1	Dengxi Opera	6	9	31	10		56
2	Tibetan Opera		12			19	31
3	Nuo Opera	4	4	19	1		28
4	Sichuan Opera	12	12				24
5	Duangong Opera		2	1	10		13
6	Yangxi Opera	3	3	7			13
7	Di Opera			11			11
8	Puppet Show	1	5	3			9
9	Shadow Puppetry	1	7		1		9
10	Others	7	10	26	13	7	63
11	Total	34	64	98	35	26	257
12	Area (1,000 km²)	82.4	486.1	176.2	394.1	1202.8	2341.6
13	Density (item/1,000 km²)	0.41	0.13	0.56	0.09	0.02	0.11

(1) **Quantity Distribution: Significant Provincial Agglomeration Gradient and Prominent Core Hosting Areas**

In terms of absolute quantity, the 257 traditional drama ICH items in Southwest China are extremely unevenly distributed across the five provincial-level regions, showing a clear quantitative gradient. Guizhou has an absolute advantage with 98 items (38.1% of the total), making it the core hosting province of traditional drama ICH in Southwest China. Sichuan is the secondary supporting area with 64 items (24.9%). Chongqing (34 items, 13.2%) and Yunnan (35 items, 13.6%) are similar in scale, while Xizang is the weakest area with 26 items (10.1%). The overall pattern is “Guizhou leading, Sichuan following, Chongqing and Yunnan in the middle, and Xizang scarce”, accompanied by an obvious spatial pattern of “dense in the east and sparse in the west”, highlighting Guizhou’s core status in regional ICH inheritance.

(2) **Density Distribution: Sharp Density Differences of Traditional Drama ICH, Characterized by “High Density in Small Provinces and Low Density in Large Provinces”**

The distribution density of traditional drama ICH varies significantly among provincial-level regions. Guizhou ranks first with a density of 0.56 items per 1,000 km², followed by Chongqing with 0.41 items per 1,000 km². Both are small in area and densely populated, forming an ICH-enriched pattern of “high density in small provinces”. The densities of Sichuan (0.13), Yunnan (0.09), and Xizang (0.02) decrease in turn. Xizang, with a vast territory of 1.2028 million km² and a scattered population, has a density only 1/28 that of Guizhou, fully reflecting that traditional drama ICH is highly dependent on population agglomeration, cultural spheres and regional living scenarios, which is distinctly different from the density law of overall ICH driven by natural water systems and urbanization [17,18].

(3) **Type Distribution: Strong Regional Specificity of Traditional Drama Types and Close Bonding with Ethnic and Regional Culture**

**Fig 3:**
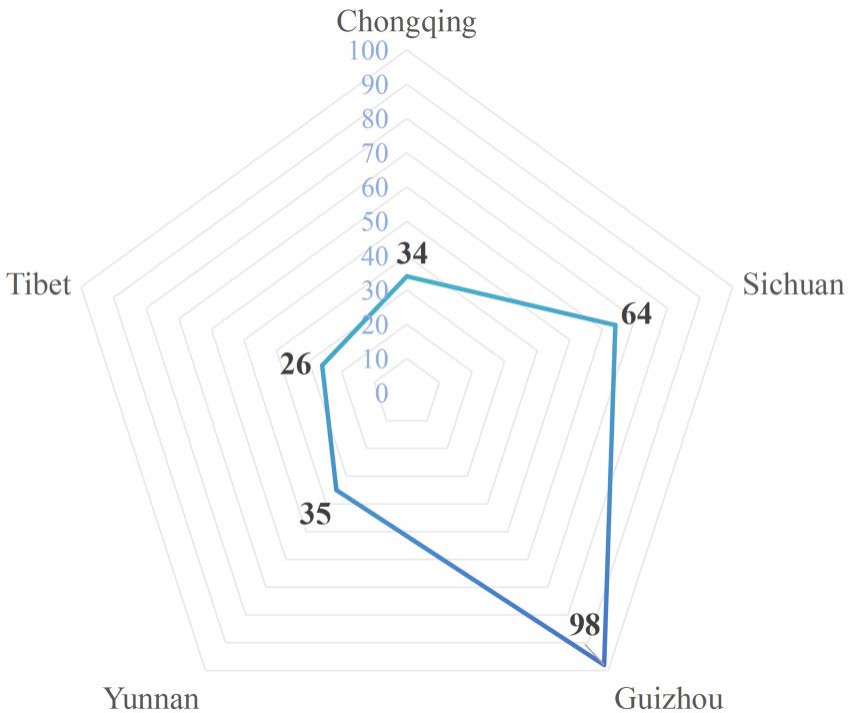
Quantity distribution of traditional drama in Southwest China.

**Fig 4:**
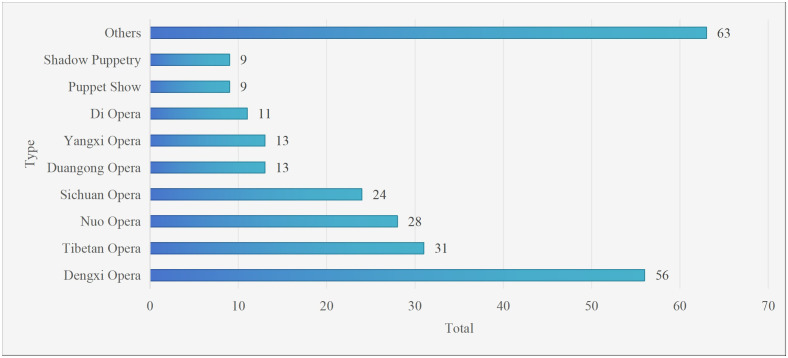
Type distribution of traditional drama ICH in Southwest China.

From the perspective of typological structure, traditional drama ICH in Southwest China presents a differentiation feature of “deep integration of type agglomeration and regional characteristics”. Dengxi Opera, with 56 items, is the largest type in the region, of which 31 items are concentrated in Guizhou, accounting for 55.4% of this type, making it the core representative ICH type of Guizhou. The 31 items of Tibetan Opera are highly agglomerated in Xizang (19 items) and Sichuan (12 items), showing extremely strong ethnic and regional specificity and serving as the iconic ICH of the Qinghai-Tibet Plateau cultural circle. Among the 28 items of Nuo Opera, 19 are distributed in Guizhou, while all 24 items of Sichuan Opera are concentrated in Sichuan and Chongqing, forming a cross-regional cultural inheritance belt. The 11 items of Di Opera are distributed only in Guizhou, and types such as Puppet Show and Shadow Puppetry are scattered. The “Others” category, with 63 items, highlights the diversity and richness of traditional drama ICH types in Southwest China. Overall, the distribution of each type is highly matched with the provincial cultural endowments, forming core agglomeration highlands such as Guizhou, Sichuan and Chongqing, while retaining ethnic characteristic types in regions such as Xizang. It reflects a distinctive exclusive and regional distribution pattern relying on ethnic culture, local customs and historical inheritance (Figs 3 and 4).

### 3.3 Spatial distribution patterns of different traditional drama types

Based on the nearest neighbor index analysis (Table 2), significant differences exist in the spatial distribution patterns among different types of traditional drama ICH in Southwest China, while the overall distribution shows a strong clustering characteristic. The nearest neighbor ratio of all traditional drama ICH is 0.37, with an extremely significant z-score of −19.26 (p = 0.00), indicating that traditional drama ICH in Southwest China presents a highly clustered distribution at the overall level.

**Table 2:** Nearest neighbor index of traditional drama ICH in Southwest China.

Type	Mean Observed Distance (km)	Expected Mean Distance (km)	Nearest Neighbor Ratio	z-Score	p-Value	Pattern
Dengxi Opera	23.72	49.98	0.47	−7.52	0.00	Clustered
Tibetan Opera	67.31	95.67	0.70	−3.16	0.00	Clustered
Nuo Opera	69.57	62.63	1.11	1.12	0.26	Random
Sichuan Opera	45.37	37.26	1.21	2.04	0.04	Dispersed
Duangong Opera	46.38	59.33	0.78	−1.51	0.13	Random
Yangxi Opera	64.65	75.47	0.86	−0.99	0.32	Random
Di Opera	21.73	14.67	1.48	3.05	0.00	Dispersed
Puppet Show	55.81	56.15	0.99	−0.03	0.97	Random
Shadow Puppetry	150.29	107.13	1.40	2.31	0.02	Dispersed
Others	59.52	89.23	0.66	−5.05	0.00	Clustered
All Types	20.38	54.80	0.37	−19.26	0.00	Clustered

(1) **Clustered distribution types**: Dengxi Opera, Tibetan Opera, and the “Others” category show significant clustering. Dengxi Opera has the lowest ratio of 0.47, representing the strongest clustering intensity. Tibetan Opera (ratio = 0.70) also shows stable and significant clustering. Notably, the “Others” type, which includes 63 kinds of small local and ethnic small operas, has a nearest neighbor ratio of 0.66 (p = 0.00), showing a strong spatial clustering feature. This indicates that most small traditional dramas are not randomly scattered, but are intensively inherited in specific ethnic settlements, mountainous areas and small cultural circles, which reflects the close dependence of small drama types on regional cultural ecology.
(2) **Random distribution types**: Nuo Opera, Duangong Opera, Yangxi Opera, and Puppet Show show random spatial distribution. Their nearest neighbor ratios are close to 1.00, and the p-values are all greater than 0.05, showing no obvious clustering or discrete trend, with relatively flexible inheritance regions.
(3) **Dispersed distribution types**: Sichuan Opera, Di Opera, and Shadow Puppetry present significant dispersed patterns. Di Opera has the highest dispersion degree (ratio = 1.48), followed by Shadow Puppetry (1.40) and Sichuan Opera (1.21), all statistically significant (p < 0.05), indicating a relatively wide spread scope and strong regional mobility.

In summary, the spatial distribution of traditional drama ICH in Southwest China is dominated by clustering, supplemented by random and dispersed patterns. The strong clustering of the “Others” category further confirms that the inheritance of small local and ethnic dramas is deeply embedded in specific regional cultural spaces, which is an important supplement to the overall agglomeration pattern of traditional drama ICH.

### 3.4 Temporal evolution and spatial migration of traditional drama ICH

**Fig 5:**
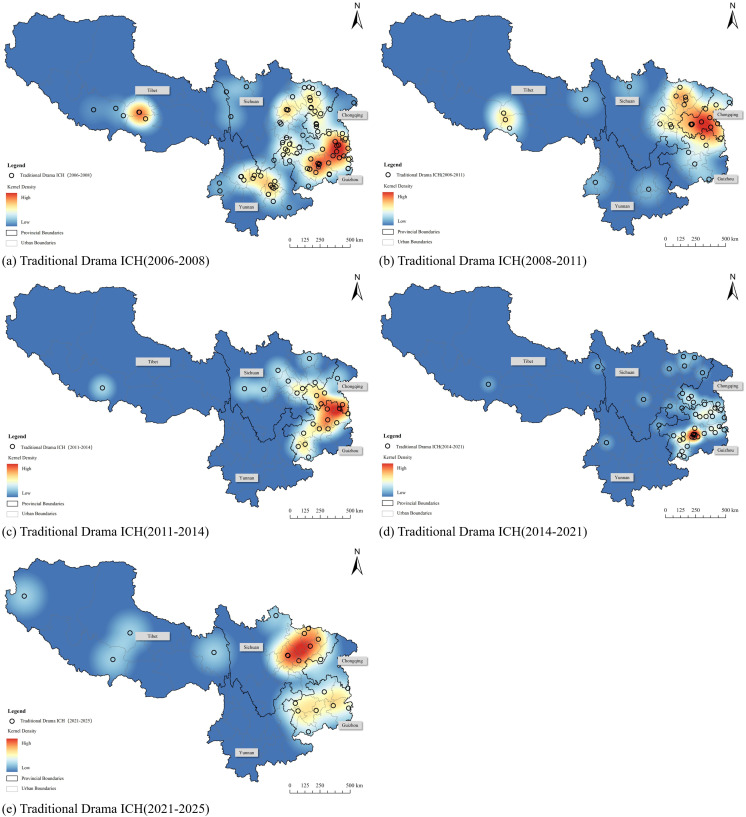
Kernel density analysis of traditional drama ICH in Southwest China by batch.

**Fig 6:**
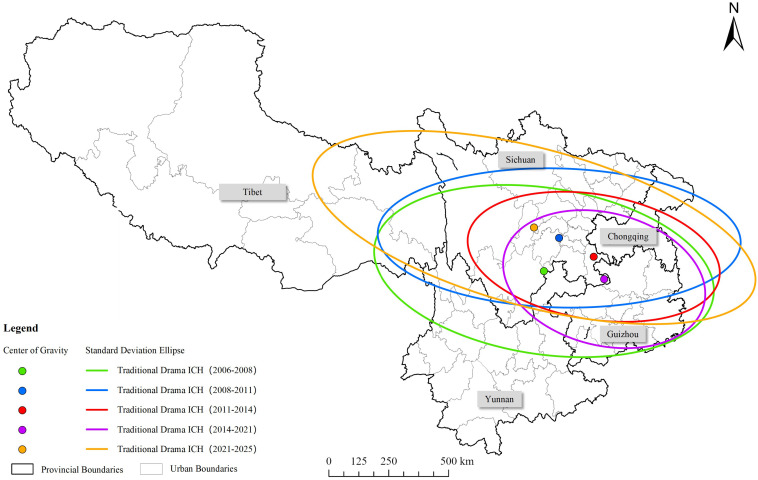
Standard deviation ellipse analysis of the traditional drama ICH protection process in Southwest China.

This study divides the research interval from 2006 to 2025 into five consecutive stages by applying the natural break point method, which is fully consistent with the release sequence of the five batches of national-level ICH lists and the key nodes of provincial-level ICH declaration in the five provinces of Southwest China. The five stages are defined as: 2006–2008 (initial announcement stage), 2008–2011 (initial expansion stage), 2011–2014 (stable development stage), 2014–2021 (comprehensive promotion stage), and 2021–2025 (high-quality agglomeration stage). Such a classification conforms to the national policy system of ICH protection, accurately identifies the critical nodes of quantitative growth and spatial evolution of traditional drama ICH, and ensures the scientificity and rationality of the temporal–spatial analysis [19]. On this basis, combined with kernel density estimation (Fig 5) and standard deviational ellipse analysis (Fig 6) for the five periods, this section systematically explores the temporal evolution characteristics and spatial migration laws of traditional drama ICH in Southwest China.

#### 3.4.1 Temporal evolution of spatial agglomeration patterns.

From the perspective of kernel density distribution, the spatial agglomeration pattern of traditional drama ICH in Southwest China has shown a dynamic evolution process of “multi-core initial formation, core migration, and continuous concentration” over the past 20 years:

- **2006–2008 (Initial Stage)**: The agglomeration pattern was initially formed, with two high-density cores in central-eastern Guizhou and central Sichuan, and scattered agglomeration points in Xizang, Yunnan, and other regions. The overall distribution was relatively scattered, and the agglomeration intensity was weak, laying the foundation for the spatial pattern of traditional drama ICH in Southwest China.
- **2008–2011 (Core Strengthening Stage)**: The high-density core shifted to the Chongqing-Guizhou border, with a significant increase in agglomeration intensity, forming a single strong core in the east. The agglomeration points in Xizang and Yunnan were further concentrated, and the spatial pattern of “dense in the east and sparse in the west” began to take shape.
- **2011–2014 (Core Diffusion Stage)**: The high-density core remained in the Chongqing-Guizhou border, and the agglomeration scope expanded to the surrounding areas. At the same time, a secondary agglomeration zone appeared in southern Guizhou, and the distribution scope of traditional drama ICH further expanded.
- **2014–2021 (Multi-point Spread Stage)**: The high-density core shifted to central Guizhou, and the agglomeration intensity decreased, showing a trend of multi-point spread. The number of agglomeration points in Sichuan, Chongqing, and Guizhou increased significantly, and the distribution became more balanced.
- **2021–2025 (High Concentration Stage)**: The high-density core returned to the Sichuan-Chongqing border, forming a super-high agglomeration zone with extremely high intensity. The overall distribution showed a trend of further concentration to the core area, and the spatial pattern of “one core with multiple points” was finally formed.

#### 3.4.2 Spatial migration of the center of gravity and standard deviation ellipse.

From the perspective of the center of gravity and standard deviation ellipse, the spatial distribution center of traditional drama ICH in Southwest China has shown a “southwest-northeast-southwest” migration trajectory, and the distribution scope has shown a trend of “first shrinking, then expanding, and then shrinking”:

(1) **Center of Gravity Migration**: The center of gravity of traditional drama ICH was initially located in northeastern Guizhou (2006–2008), then moved to northern Guizhou (2008–2011), then to the Chongqing-Guizhou border (2011–2014), then to central Guizhou (2014–2021), and finally returned to the Sichuan-Chongqing border (2021–2025). The overall migration direction was from Guizhou to the Sichuan-Chongqing border, reflecting the continuous strengthening of the core position of the Sichuan-Chongqing-Guizhou cultural integration area in the inheritance of traditional drama ICH.
(2) **Standard Deviation Ellipse Change**: The standard deviation ellipse showed a trend of “first shrinking, then expanding, and then shrinking”. The ellipse in 2006–2008 was the smallest, indicating the highest initial concentration; the ellipse in 2021–2025 was the largest, indicating the widest distribution scope; the ellipse in 2014–2021 shrank again, reflecting the re-concentration of traditional drama ICH to the core area. The long axis of the ellipse was always in the northeast-southwest direction, indicating that the spatial distribution of traditional drama ICH in Southwest China has always maintained a northeast-southwest directional distribution pattern, which is highly coupled with the geographical pattern of the Sichuan-Chongqing-Guizhou border.

#### 3.4.3 Overall evolution law and driving mechanism.

The spatial distribution of traditional drama ICH in Southwest China from 2006 to 2025 has shown a dynamic evolution process of “from scattered to concentrated, from multi-core to single core, and from Guizhou-led to Sichuan-Chongqing-Guizhou co-led”. The continuous migration of the center of gravity and the change of the standard deviation ellipse fully reflect the dynamic adjustment of the inheritance pattern of traditional drama ICH in Southwest China under the influence of policies, economy, culture and other factors. The core position of the Sichuan-Chongqing-Guizhou border has been continuously strengthened, which is the result of the joint action of the profound cultural heritage, dense population, and convenient cultural exchange in this region, and also reflects the important role of regional cultural integration in the protection and inheritance of traditional drama ICH [15,18].

## 4. Influencing factors of the spatial distribution of traditional drama ICH

Based on the geographical detector model, this section examines the single-factor explanatory power and interactive influence of eight selected factors covering economic development, natural resources, and cultural factors [16,20–22]. The results are shown in Tables 3 and 4, revealing the dominant driving mechanism behind the spatial distribution of traditional drama ICH in Southwest China.

**Table 3:** Results of factor detection.

Dimension	Factor	Interpretation	Data Source	q-Value	p-Value
**Economic Development**	GDP	Core indicator measuring regional economic scale and activity	Statistical Yearbook	0.0472	0.2185
	Urbanization	Directly measures regional urbanization process and population concentration	Statistical Yearbook	0.0031	0.9913
	Nighttime Light Index	Nighttime light intensity is closely related to economic activity, population density, etc., indirectly reflecting economic development level	CAS Luojia-01 NTL Data	0.0480	0.0175
	Transportation	Transportation network density, capacity, etc., are important supports for regional economic development and population flow	Statistical Yearbook	0.0971	<0.01
**Natural Resources**	Surface Water	Water resource distribution and abundance significantly impact regional development and population distribution	National Geomatics Center	0.0301	0.3317
	Terrain Elevation	Altitude affects climate, ecology, transportation, etc., thereby influencing regional development patterns	Geospatial Data Cloud	0.3029	<0.01
**Cultural Factors**	Number of Traditional Villages	The total quantity of historically and culturally protected villages in a region	Statistical Yearbook	0.1381	<0.01
	Cultural Institutions	Number of cultural institutions (museums, libraries, theaters) reflects regional cultural development level and public cultural service capacity	Statistical Yearbook	0.1153	<0.01

**Table 4:** Results of interaction between different influencing factors.

Factor	GDP	Urbanization	Surface Water	Terrain Elevation	Night Light Index	Number of Traditional Villages	Transportation	Cultural Institutions
GDP								
Urbanization	0.1363							
Surface Water	0.1221	0.0737						
Terrain Elevation	0.3982	0.3773	0.3681					
Night Light Index	0.1215	0.1017	0.1191	0.3876				
Number of Traditional Villages	0.1509	0.1572	0.1502	0.3862	0.1562			
Transportation	0.2171	0.1643	0.1687	0.4148	0.1835	0.2206		
Cultural Institutions	0.2150	0.2241	0.2493	0.4296	0.1897	0.2285	0.3287	

### 4.1 Single-factor detection analysis

The q-statistic from the factor detector reflects the explanatory power of each influencing factor [23]. A higher q-value indicates a stronger influence on the spatial distribution of traditional drama ICH [14,24]. According to Table 3, the eight factors differ significantly in their explanatory power, which can be ranked as follows: Terrain Elevation> Number of Traditional Villages> Cultural Institutions> Transportation> Nighttime Light Index> GDP> Surface Water> Urbanization.

(1) **Natural factors:** Terrain Elevation has the strongest explanatory power with a q-value of 0.3029 (p < 0.01), indicating that altitude is the dominant natural constraint shaping the spatial layout of traditional drama ICH. Surface Water shows a weak effect (q = 0.0301, p = 0.3317), which is not statistically significant.
(2) **Cultural factors:** Both Number of Traditional Villages (q = 0.1381, p < 0.01) and Cultural Institutions (q = 0.1153, p < 0.01) present strong and significant explanatory power, revealing that traditional cultural carriers and public cultural services are key supporting conditions for the inheritance of traditional drama.
(3) **Economic and social factors:** Transportation (q = 0.0971, p < 0.01) and Nighttime Light Index (q = 0.0480, p = 0.0175) exert significant but relatively weak impacts. GDP (q = 0.0472, p = 0.2185) and Urbanization (q = 0.0031, p = 0.9913) show non-significant effects, indicating that general economic scale and urbanization level are not decisive drivers.

### 4.2 Interaction detection analysis

As shown in Table 4, the interaction detector demonstrates that all two-factor combinations present significantly enhanced explanatory power compared with individual factors, mainly in the forms of bivariate enhancement and non-linear enhancement [25,26]. No independent or weakening interaction is detected [27]. The strongest interactive effect appears between Terrain Elevation and Cultural Institutions (q = 0.4296), followed by Terrain Elevation and Transportation (q = 0.4148), and Terrain Elevation and Nighttime Light Index (q = 0.3876). High-strength interactions also occur between terrain and cultural factors such as Terrain Elevation and Number of Traditional Villages (q = 0.3862), confirming that the spatial distribution of traditional drama ICH is jointly dominated by topographic conditions and cultural endowment. Interactions among cultural and economic factors, including Number of Traditional Villages with Transportation or Cultural Institutions, also produce obvious synergistic effects, which further stabilize the spatial agglomeration pattern.

### 4.3 Comprehensive driving mechanism

The spatial distribution of traditional drama ICH in Southwest China is shaped by a combined multi-factor driving system. Terrain elevation acts as the dominant natural constraint with the strongest independent explanatory power, while number of traditional villages and cultural institutions serve as core cultural factors that stably support the inheritance and agglomeration of traditional drama. Transportation and nighttime light index provide auxiliary social and economic driving forces, whereas GDP, urbanization, and surface water have limited independent impacts but contribute significantly through interactive enhancement. The interaction of any two factors produces a bivariate or non-linear enhancement effect [15], and the strongest synergy occurs between terrain elevation and cultural institutions, indicating that the spatial pattern of traditional drama ICH is not determined by a single factor but jointly dominated by natural conditions, cultural carriers, and socioeconomic conditions. Together, these factors form the comprehensive driving mechanism that underlies the “dense in the east and sparse in the west” agglomeration structure of traditional drama ICH in Southwest China.

## 5. Conclusions and discussion

### 5.1 Conclusions

This study takes 257 traditional drama intangible cultural heritage (ICH) items in Southwest China as research objects, including 54 national-level and 203 provincial-level items. By adopting GIS spatial analysis methods such as kernel density estimation, nearest neighbor index, standard deviational ellipse, and geographical detector model, this paper systematically reveals the spatial-temporal distribution characteristics, type differentiation, evolution laws and driving mechanism of traditional drama ICH in Southwest China. The main conclusions are as follows:

(1) The overall spatial distribution of traditional drama ICH in Southwest China presents a significant agglomeration pattern characterized by “dense in the east and sparse in the west, multi-core agglomeration and gradient decline”. High-density cores are concentrated in eastern Guizhou, the border area of Sichuan-Chongqing-Guizhou, while western Sichuan, western Yunnan and Xizang are sparsely distributed. The provincial distribution is extremely uneven: Guizhou ranks first with 98 items, followed by Sichuan (64 items), Yunnan (35 items), Chongqing (34 items) and Xizang (26 items). In terms of distribution density, Guizhou and Chongqing show the feature of “small provinces with high density”, while Xizang has the lowest density due to its vast territory and sparse population.
(2) Different types of traditional drama ICH have obvious spatial differentiation. Dengxi Opera, Tibetan Opera and the “Others” category (small local operas) are significantly clustered; Nuo Opera, Duangong Opera, Yangxi Opera and Puppet Show are randomly distributed; Sichuan Opera, Di Opera and Shadow Puppetry are discretely distributed. Among them, the “Others” type (63 items) also presents a strong clustering feature, indicating that most small and ethnic dramas rely on specific cultural spaces for inheritance.
(3) From 2006 to 2025, the temporal and spatial evolution of traditional drama ICH is divided into five stages based on the natural break point method and the release rhythm of national and provincial ICH lists. The overall evolution shows a law of “initial scattering → core strengthening → diffusion → multi-point expansion → high-quality agglomeration”. The center of gravity migrates within the Sichuan-Chongqing-Guizhou border area, and the standard deviational ellipse always maintains a northeast-southwest direction, highly coupled with the regional cultural corridor.
(4) The geographical detector results show that the spatial distribution of traditional drama ICH is affected by the combined effects of natural, cultural and social-economic factors. Terrain elevation (q = 0.3029) is the dominant natural constraint; number of traditional villages (q = 0.1381) and cultural institutions (q = 0.1153) are core cultural driving forces; transportation and nighttime light index play auxiliary roles, while GDP and urbanization have no significant independent impact. All two-factor interactions show an enhanced effect, and the strongest synergy is between terrain elevation and cultural institutions (q = 0.4296).

### 5.2 Discussion

Traditional drama ICH is a typical living performing heritage with fluidity, mobility and scenario-dependent inheritance [24,25]. Different from tangible heritage such as ancient buildings and ruins, its spatial distribution should not be understood as fixed physical sites, but as a set of spatial anchors rooted in regional cultural ecology, population gathering, and living transmission scenarios [28]. In this study, the “spatial location” of traditional drama ICH refers to the main inheritance community, representative protection unit, or core cultural area recorded in the official ICH system, rather than a single immobile place where the heritage exists. This operationalization allows regional GIS analysis while recognizing the mobile, event-based, and community-dependent nature of performing ICH. Therefore, this study responds to the academic concern that “spatial positioning of ICH is disconnected from its essential attributes” by constructing a spatial analysis framework based on core inheritance areas and main activity places, which retains the operability of GIS analysis while conforming to the fluid transmission logic of traditional drama.

This interpretation also places the Chinese case in dialogue with international scholarship on living heritage and performing arts spaces. Similar to studies that regard cultural landscapes as networks of memory, planning, and community use [9,10], the spatial pattern identified in this paper should be understood as an inherited cultural field rather than a closed administrative container. In addition, the discussion of live music spaces shows that performance can continuously create and reinterpret place through repeated events and social participation [28]. The present study echoes this view by treating the official location of each drama item as a spatial anchor of transmission, while acknowledging that the actual cultural practice may extend across villages, markets, festivals, theaters, and interregional performance routes.

The results confirm that cultural carriers and terrain conditions dominate the spatial pattern of traditional drama. The number of traditional villages directly reflects the integrity of cultural space and the continuity of folk customs, which is the most critical carrier for the survival of rural and ethnic dramas. This finding is consistent with the viewpoint of cultural ecological theory and living heritage protection: the existence and inheritance of ICH are highly dependent on its cultural space and community environment [29,30]. Meanwhile, terrain elevation restricts population distribution and traffic accessibility, forming the basic pattern of “dense in eastern section and sparse in western section”, which echoes the research conclusions of ICH spatial distribution in mountainous and plateau areas.

It is worth noting that the “Others” category (small dramas) shows a strong clustering feature, which reveals that a large number of small-scale, regional and ethnic dramas are concentrated in specific settlements and cultural circles. They are important components of regional cultural diversity but are often ignored in research. This reminds us that the protection of traditional drama ICH should not only focus on large and well-known types but also pay attention to the living protection of small dramas embedded in traditional villages and ethnic areas.

In terms of driving factors, the non-significant effect of GDP and urbanization indicates that economic development does not directly determine the inheritance of traditional drama. Instead, public cultural services (cultural institutions), traditional space carriers and convenient transportation are more critical. This provides an important implication for protection practice: the inheritance of traditional drama relies more on cultural ecology and public welfare support than pure economic input [22]. The interaction enhancement effect further shows that the protection of traditional drama ICH needs the coordination of natural suitability, cultural space and social services.

From the perspective of interdisciplinary integration, this study can be further connected with cultural neuroscience, therapeutic arts and digital humanities [31]. As Mastnak’s research pointed out, traditional drama, as a performing art, has the function of shaping cultural memory, emotional resonance and community identity, and can be used as a kind of “cultural therapy” to enhance community cohesion and mental health [32,33]. At the same time, combined with the STEMAC concept [31], digital means such as VR/AR, digital archives and AI can be used to realize the digital protection and living inheritance of drama.

We acknowledge several limitations in this study. The spatial positioning adopts the core inheritance area as the coordinate, which cannot fully reflect the cross-regional flow and transmission network of drama. The influencing factors do not include policy systems and inheritance mechanisms. Construction of a fuller formation-and- influence mechanism framework could be an important direction for future research. In particular, future comparative studies could examine whether the interaction among cultural carriers, accessibility, and public cultural services observed in Southwest China also appears in European traditional drama, Southeast Asian folk performing arts, or other community-based performing traditions. Such comparison would help distinguish context-specific Chinese policy effects from more general spatial conditions of living heritage transmission.

### 5.3 Implications for protection and policy

Combined with the spatial-temporal pattern and driving mechanism, this study puts forward three-level protection implications:

(1) **Build a hierarchical protection system**: Focus on protecting high-density agglomeration areas such as eastern Guizhou and Sichuan-Chongqing-Guizhou border, and carry out regional overall protection; strengthen the targeted protection of small dramas in the “Others” category to maintain cultural diversity.
(2) **Optimize the cultural ecological environment**: Take traditional villages as the core carrier, link cultural institutions and transportation facilities, and build a “village + drama + culture” integrated inheritance space.
(3) **Strengthen interdisciplinary and digital protection:** Introduce digital technologies such as virtual reality/augmented reality (VR/AR) and digital archives to achieve the dynamic inheritance of traditional drama; explore the social and therapeutic value of traditional drama and expand its application scenarios in community governance and cultural identity.

## Supporting information

S1 File The full list of traditional drama ICH in Southwestern China. (DOCX)
