# Supplementary material for: Uneven development of intangible cultural heritage: A spatial analysis of traditional drama in Southwestern China
Source: PLoS One. 2026 Aug 14;21(8):e0355102. doi: 10.1371/journal.pone.0355102 (PMC13475970; doi:10.1371/journal.pone.0355102)
Supplement: Supplementary file 1 [file pone.0355102.s001.docx]

**S1 File. The full list of traditional drama ICH in Southwestern China**

| **Province & No.** | **Name** | **Level** | **Type** | **County** | **Year of announcement** |
| --- | --- | --- | --- | --- | --- |
| Chongqing-1 | Sichuan Opera | National Level | Sichuan Opera | Chongqing | 2006 |
| Chongqing-2 | Dengxi Opera（Liangshan Dengxi Opera） | National Level | Dengxi Opera | Liangping County, Chongqing | 2006 |
| Chongqing-3 | Yangxi Opera（The Tujia Masked Yangxi Opera of Youyang） | National Level | Yangxi Opera | Yuyang Tujia and Miao Autonomous County, Chongqing | 2021 |
| Chongqing-4 | Sichuan Opera | Provincial Level | Sichuan Opera | Sichuan Opera Institute, Chongqing | 2007 |
| Chongqing-5 | Liangshan Dengxi Opera | Provincial Level | Dengxi Opera | Liangping County | 2007 |
| Chongqing-6 | Masked Yangxi Opera | Provincial Level | Yangxi Opera | Youyang County | 2007 |
| Chongqing-7 | Jielong Nuo Opera | Provincial Level | Nuo Opera | Banan District | 2007 |
| Chongqing-8 | Yangxi Opera | Provincial Level | Yangxi Opera | Xiushan County | 2007 |
| Chongqing-9 | Yujia Nuo Opera | Provincial Level | Nuo Opera | Xiushan County | 2007 |
| Chongqing-10 | Shihaoyang Opera | Provincial Level | Others | Qijiang County | 2007 |
| Chongqing-11 | Caitang Opera | Provincial Level | Others | Wushan County | 2007 |
| Chongqing-12 | Sichuan-Chongqing Puppet Show | Provincial Level | Puppet Show | Yuzhong District | 2009 |
| Chongqing-13 | Li Family School of Peking Opera Art | Provincial Level | Others | City Opera Troupe | 2009 |
| Chongqing-14 | Shadow puppetry in the Three Gorges area | Provincial Level | Shadow Puppetry | Wushan County | 2009 |
| Chongqing-15 | Stone Pillar Folk Opera | Provincial Level | Others | Shizhu County | 2009 |
| Chongqing-16 | Chenhe Opera | Provincial Level | Others | Xiushan County | 2009 |
| Chongqing-17 | Baoandenger Opera | Provincial Level | Dengxi Opera | Xiushan County | 2009 |
| Chongqing-18 | Mulazhuang Nuo Opera | Provincial Level | Nuo Opera | Pengshui County | 2009 |
| Chongqing-19 | Zhuoshui Houxhe Opera | Provincial Level | Others | Qianjiang District | 2009 |
| Chongqing-20 | Liangping Nuo Opera | Provincial Level | Nuo Opera | Liangping County | 2009 |
| Chongqing-21 | Sichuan Opera and Sichuan Opera Music | Provincial Level | Sichuan Opera | Shapingba District | 2009 |
| Chongqing-22 | Sichuan Opera | Provincial Level | Sichuan Opera | Jiulongpo District | 2011 |
| Chongqing-23 | Yaya Opera | Provincial Level | Others | Yunyang County | 2011 |
| Chongqing-24 | Sichuan Opera | Provincial Level | Sichuan Opera | Yubei District | 2014 |
| Chongqing-25 | Sichuan Opera | Provincial Level | Sichuan Opera | Wansheng Economic Development Zone | 2014 |
| Chongqing-26 | Youyang Dengxi Opera | Provincial Level | Dengxi Opera | Youchang County | 2014 |
| Chongqing-27 | Sichuan Opera | Provincial Level | Sichuan Opera | Wanzhou District | 2016 |
| Chongqing-28 | Fuzhou Sichuan Opera | Provincial Level | Sichuan Opera | Fuling District | 2016 |
| Chongqing-29 | Baogu Dengxi Opera | Provincial Level | Dengxi Opera | Youyang County | 2016 |
| Chongqing-30 | Sichuan Opera | Provincial Level | Sichuan Opera | Dadukou District | 2019 |
| Chongqing-31 | Sichuan Opera | Provincial Level | Sichuan Opera | Jiangjin District | 2019 |
| Chongqing-32 | Sichuan Opera | Provincial Level | Sichuan Opera | Qijiang District | 2019 |
| Chongqing-33 | Sichuan Opera | Provincial Level | Sichuan Opera | Dazu District | 2019 |
| Chongqing-34 | Xiushanhua Dengxi Opera | Provincial Level | Dengxi Opera | Xiushan County | 2019 |
| Sichuan-1 | Sichuan Opera | National Level | Sichuan Opera | Sichuan Province | 2006 |
| Sichuan-2 | Dengxi Opera（Chuanbei Dengxi Opera） | National Level | Dengxi Opera | Nanchong City, Sichuan | 2006 |
| Sichuan-3 | Tibetan Opera（Degegesaer Tibetan Opera） | National Level | Tibetan Opera | Dege County, Sichuan | 2008 |
| Sichuan-4 | Tibetan Opera（Batang Tibetan Opera） | National Level | Tibetan Opera | Batao County, Sichuan | 2008 |
| Sichuan-5 | Tibetan Opera（Seda Tibetan Opera） | National Level | Tibetan Opera | Seda County, Sichuan | 2008 |
| Sichuan-6 | Shadow Puppetry（Sichuan Shadow Puppetry） | National Level | Shadow Puppetry | Lanzhong City, Sichuan Province | 2008 |
| Sichuan-7 | Shadow Puppetry（Sichuan Shadow Puppetry） | National Level | Shadow Puppetry | Southern County, Sichuan Province | 2008 |
| Sichuan-8 | Puppet Show（Chuanbei Puppet Show） | National Level | Puppet Show | Sichuan Province | 2006 |
| Sichuan-9 | Puppet Show（Medium-sized Stick Puppet Show） | National Level | Puppet Show | Zizhong County, Sichuan Province | 2014 |
| Sichuan-10 | Yangxi Opera（Shejianti Yangxi Opera） | National Level | Yangxi Opera | Zhaohua City, Guangyuan Prefecture, Sichuan Province | 2014 |
| Sichuan-11 | Duangong Opera（Wangcang Duangong Opera） | National Level | Duangong Opera | Guangyuan City, Sichuan Province | 2021 |
| Sichuan-12 | Sichuan Opera | Provincial Level | Sichuan Opera | Provincial-level institutions | 2007 |
| Sichuan-13 | Sichuan Opera（Luozhou River Sichuan Opera） | Provincial Level | Sichuan Opera | Luzhou City | 2007 |
| Sichuan-14 | Sichuan Shadow Puppetry (Chengdu） | Provincial Level | Shadow Puppetry | Chengdu City | 2007 |
| Sichuan-15 | Sichuan Shadow Puppetry（Gaoguan Shadow Puppetry） | Provincial Level | Shadow Puppetry | GuanYang City, JianGe County | 2007 |
| Sichuan-16 | Sichuan Shadow Puppetry（Bazhong Shadow Puppetry） | Provincial Level | Shadow Puppetry | Bazhong City, BaZhou District | 2007 |
| Sichuan-17 | Sichuan Shadow Puppetry（Langzhong Shadow Puppetry） | Provincial Level | Shadow Puppetry | Nanchong City, LangZhen City | 2007 |
| Sichuan-18 | Sichuan Shadow Puppetry（The He Family Shadow Puppetry） | Provincial Level | Shadow Puppetry | Nanchong City, Nanbu County | 2007 |
| Sichuan-19 | Dengxi Opera（Xu Jiawan's Twelve Flowers Dengxi Opera） | Provincial Level | Dengxi Opera | Mianyang City, BeiChuan Qiang Autonomous County | 2007 |
| Sichuan-20 | Dengxi Opera（Emei Hall Dengxi Opera） | Provincial Level | Dengxi Opera | Emao Mountain City, Leshan City | 2007 |
| Sichuan-21 | Dengxi Opera（Jiajiang Malaotang Dengxi Opera） | Provincial Level | Dengxi Opera | Jiajiang County, Leshan City | 2007 |
| Sichuan-22 | Dengxi Opera（Yuechi Dengxi Opera） | Provincial Level | Dengxi Opera | Yuechi County, Guang'an City | 2007 |
| Sichuan-23 | Dengxi Opera（Chuanbei Dengxi Opera） | Provincial Level | Dengxi Opera | Nanchong City | 2007 |
| Sichuan-24 | Puppet Show（Chengdu Puppet Show） | Provincial Level | Puppet Show | Chengdu City | 2007 |
| Sichuan-25 | Puppet Show（Palm Puppet Show） | Provincial Level | Puppet Show | Linshui County, Guang'an City | 2007 |
| Sichuan-26 | Puppet Show（Chuanbei Puppet Show） | Provincial Level | Puppet Show | Nanchong City | 2007 |
| Sichuan-27 | Shejianti Yangxi Opera | Provincial Level | Yangxi Opera | Zhaohua District, Guangyuan City | 2007 |
| Sichuan-28 | Wangcang Duangong Opera | Provincial Level | Duangong Opera | Wangcang County, Guangyuan City | 2007 |
| Sichuan-29 | Tibetan Opera（Dege Tibetan Opera） | Provincial Level | Tibetan Opera | Zixiong County, Mianyang City | 2007 |
| Sichuan-30 | Tibetan Opera（Batang Tibetan Opera） | Provincial Level | Tibetan Opera | Qingyang District, Chengdu City | 2007 |
| Sichuan-31 | Tibetan Opera（Seda Tibetan Opera） | Provincial Level | Tibetan Opera | Jiange County, Guangyuan City | 2007 |
| Sichuan-32 | Maming Yangxi Opera | Provincial Level | Yangxi Opera | Nanxiong County, Nanchong City | 2009 |
| Sichuan-33 | The Bed Sheet Opera | Provincial Level | Others | Leshan City | 2009 |
| Sichuan-34 | White Dragon Paper Figure | Provincial Level | Others | Guangyuan City Jiange County | 2009 |
| Sichuan-35 | Southern Nuo Opera | Provincial Level | Nuo Opera | Nanchong City Nanbu County | 2009 |
| Sichuan-36 | Nanmuda Tibetan Opera | Provincial Level | Tibetan Opera | Leshan City | 2009 |
| Sichuan-37 | Sichuan Opera（Jiayang River Sichuan Opera） | Provincial Level | Sichuan Opera | Nanchong City Langzhong City | 2009 |
| Sichuan-38 | Dengxi Opera[Chuanbei Dengxi Opera（Liangzhong LaoguanDengxi Opera）] | Provincial Level | Dengxi Opera | Zigong City | 2009 |
| Sichuan-39 | Sichuan Opera（Ziyang RiverSichuan Opera Art） | Provincial Level | Sichuan Opera | Neijiang City | 2011 |
| Sichuan-40 | Sichuan Opera（Ziyang RiverSichuan Opera Art） | Provincial Level | Sichuan Opera | Nanchong City | 2011 |
| Sichuan-41 | Sichuan Opera（Chuanbei River Sichuan Opera) | Provincial Level | Sichuan Opera | Yuechi County, Guang'an City | 2011 |
| Sichuan-42 | Qu Opera (Yuechi Qu Opera) | Provincial Level | Others | Yuechi County, Guang'an City | 2014 |
| Sichuan-43 | Qu Opera (Anyue Qu Opera) | Provincial Level | Others | Ziyang City Anyue County | 2014 |
| Sichuan-44 | Hongya Teacher's Way Opera | Provincial Level | Others | Hongya County, Meishan City | 2014 |
| Sichuan-45 | Qiang Ethnic Group's Shibi Opera | Provincial Level | Others | Litang County Cultural Center | 2014 |
| Sichuan-46 | Tibetan Opera（Muya Tibetan Opera） | Provincial Level | Tibetan Opera | Nanjiang County, Bazhong City | 2014 |
| Sichuan-47 | Tibetan Opera（Litang Tibetan Opera） | Provincial Level | Tibetan Opera | Shimen County, Ya'an City | 2014 |
| Sichuan-48 | Chuan Tan Opera | Provincial Level | Others | Yibin City | 2018 |
| Sichuan-49 | Muya Tibetan Opera“Shijielabu” | Provincial Level | Tibetan Opera | Sichang County, Shi'an City, | 2018 |
| Sichuan-50 | Sichuan Opera（Xulu River Sichuan Opera Art） | Provincial Level | Sichuan Opera | Yibin City | 2018 |
| Sichuan-51 | Sichuan Opera（Baqu River Sichuan Opera Art） | Provincial Level | Sichuan Opera | Dazhou City | 2018 |
| Sichuan-52 | Dengxi Opera（Cangxi Dengxi Opera） | Provincial Level | Dengxi Opera | Cangxi County, Guangyuan City | 2018 |
| Sichuan-53 | The Bed Sheet Opera | Provincial Level | Others | Anzhou District, Mianyang City | 2018 |
| Sichuan-54 | Nuo Opera（Luzhou Nuo Opera） | Provincial Level | Nuo Opera | Luzhou City | 2018 |
| Sichuan-55 | Peking Opera | Provincial Level | Others | Chengdu City | 2022 |
| Sichuan-56 | Yang Village Nuo Opera | Provincial Level | Nuo Opera | Jiaoguo County, Guangyuan City | 2022 |
| Sichuan-57 | Chaotianpingxi Nuo Opera | Provincial Level | Nuo Opera | Chao Tian District, Guangyuan City | 2022 |
| Sichuan-58 | Nanbuhua Dengxi Opera | Provincial Level | Dengxi Opera | Nanbu County, Nan'gang City | 2022 |
| Sichuan-59 | Sichuan Opera（Baqu River Sichuan Opera） | Provincial Level | Sichuan Opera | Linshui County, Guang'an City | 2022 |
| Sichuan-60 | Sichuan Opera（Baqu River Sichuan Opera） | Provincial Level | Sichuan Opera | Bazhao District, Baizhong City | 2022 |
| Sichuan-61 | Sichuan Opera（Ziyang River Sichuan Opera） | Provincial Level | Sichuan Opera | Lezi County, Ziyang City | 2022 |
| Sichuan-62 | Zoige Tibetan Opera | Provincial Level | Tibetan Opera | Jiuleige County, Aba Autonomous Prefecture | 2022 |
| Sichuan-63 | Han Opera | Provincial Level | Others | Guanghan City, Deyang City, | 2025 |
| Sichuan-64 | Tibetan Opera（Meva Tibetan Opera） | Provincial Level | Tibetan Opera | Hongyuan County, Aba Autonomous Prefecture | 2025 |
| Guizhou-1 | 花Dengxi Opera（Sinan Flower Dengxi Opera） | National Level | Dengxi Opera | Siniang County, Guizhou Province | 2006 |
| Guizhou-2 | Flower Dengxi Opera | National Level | Dengxi Opera | Dushan County, Guizhou Province | 2008 |
| Guizhou-3 | Flower Dengxi Opera | National Level | Dengxi Opera | Guizhou Provincial Huadengxi Opera Troupe | 2011 |
| Guizhou-4 | Dong Opera | National Level | Others | Li Ping County, Guizhou Province | 2006 |
| Guizhou-5 | Buyi Opera | National Level | Others | Che Hong County, Guizhou Province | 2006 |
| Guizhou-6 | The Yi Ethnic Group's Tuotaiji | National Level | Others | Weining Yi, Hui and Miao Autonomous County, Guizhou Province | 2006 |
| Guizhou-7 | Nuo Opera（Dejiang Nuangang Opera） | National Level | Nuo Opera | Dejiang County, Guizhou Province | 2006 |
| Guizhou-8 | Nuo Opera（Gelao Nuo Opera） | National Level | Nuo Opera | Daozhen Yi Lao Miao Autonomous County, Guizhou Province | 2008 |
| Guizhou-9 | Nuo Opera（Liping Bouyi Ethnic Group Nuo Opera） | National Level | Nuo Opera | Liping County, Guizhou Province | 2011 |
| Guizhou-10 | Nuo Opera（Qingtan） | National Level | Nuo Opera | Jinsha County, Guizhou Province | 2014 |
| Guizhou-11 | Anshun Di Opera | National Level | Di Opera | Anshun City, Guizhou Province | 2006 |
| Guizhou-12 | Puppet Show（Shiqian Puppet Show） | National Level | Puppet Show | Shiannan County, Guizhou Province | 2006 |
| Guizhou-13 | Qian Opera | National Level | Others | Guizhou Provincial Kunqu Opera Troupe | 2008 |
| Guizhou-14 | Yangxi Opera（Guizhou Yangxi Opera） | National Level | Yangxi Opera | Guizhou Province | 2021 |
| Guizhou-15 | Dong Opera | Provincial Level | Others | Liping County | 2005 |
| Guizhou-16 | Sizhou Nuo Opera | Provincial Level | Nuo Opera | Cenggong County | 2005 |
| Guizhou-17 | Fuquan Yangxi Opera | Provincial Level | Yangxi Opera | Fuquan City | 2005 |
| Guizhou-18 | Sinan Huadeng Opera | Provincial Level | Dengxi Opera | Sinan County | 2005 |
| Guizhou-19 | Dejiang Nuangang Opera | Provincial Level | Nuo Opera | Dejiang County | 2005 |
| Guizhou-20 | Shiqian Puppet Show | Provincial Level | Puppet Show | Shiqian County | 2005 |
| Guizhou-21 | Buyi Opera | Provincial Level | Others | Chehen County | 2005 |
| Guizhou-22 | Anshun Di Opera | Provincial Level | Di Opera | Anshun City | 2005 |
| Guizhou-23 | Yangxi Opera | Provincial Level | Yangxi Opera | Tianzhu County | 2007 |
| Guizhou-24 | Vincine Opera | Provincial Level | Others | Qianxi County | 2007 |
| Guizhou-25 | Vincine Opera | Provincial Level | Others | Tongren City | 2007 |
| Guizhou-26 | Vincine Opera | Provincial Level | Others | Zunyi City | 2007 |
| Guizhou-27 | Vincine Opera | Provincial Level | Others | Wudang District | 2007 |
| Guizhou-28 | Huadeng Opera | Provincial Level | Dengxi Opera | Puding County | 2007 |
| Guizhou-29 | Huadeng Opera | Provincial Level | Dengxi Opera | Dusanjia County | 2007 |
| Guizhou-30 | Huadeng Opera | Provincial Level | Dengxi Opera | Qianxi County | 2007 |
| Guizhou-31 | Huadeng Opera | Provincial Level | Dengxi Opera | Fuquan City | 2007 |
| Guizhou-32 | Huadeng Opera | Provincial Level | Dengxi Opera | Huaxi District | 2007 |
| Guizhou-33 | Huadeng Opera | Provincial Level | Dengxi Opera | Zunyi City | 2007 |
| Guizhou-34 | Huadeng Opera | Provincial Level | Dengxi Opera | Yugeng County | 2007 |
| Guizhou-35 | Huadeng Opera | Provincial Level | Dengxi Opera | Shiqian County | 2007 |
| Guizhou-36 | Huadeng Opera | Provincial Level | Dengxi Opera | Yinjiang County | 2007 |
| Guizhou-37 | Gelao Nuo Opera | Provincial Level | Nuo Opera | Daozhen County | 2007 |
| Guizhou-38 | Qian Opera | Provincial Level | Others | Qian Opera Troupe of Guizhou Provincial | 2007 |
| Guizhou-39 | Sizhou Xiannuo God Opera | Provincial Level | Nuo Opera | Cen Gong County | 2007 |
| Guizhou-40 | Zhenyuan Tujia Ethnic Group Nuo Opera | Provincial Level | Nuo Opera | Zhen Yuan County | 2007 |
| Guizhou-41 | Penglai Buyi Di Opera | Provincial Level | Di Opera | Baiyun District | 2007 |
| Guizhou-42 | Malutunbao Di Opera | Provincial Level | Di Opera | Chang Shun County | 2007 |
| Guizhou-43 | Silk-string lamp Opera | Provincial Level | Dengxi Opera | Feng Gao County | 2009 |
| Guizhou-44 | The Gela ethnic group's "Rolling Dragon Opera" | Provincial Level | Others | Zheng An County | 2009 |
| Guizhou-45 | Duangong Opera | Provincial Level | Duangong Opera | Jin Sha County | 2009 |
| Guizhou-46 | Mamadeng Opera | Provincial Level | Others | Zheng An County | 2015 |
| Guizhou-47 | Chadeng Opera | Provincial Level | Dengxi Opera | Song Tao Miao Autonomous County | 2015 |
| Guizhou-48 | The "Yanmen" of the Bouyei ethnic group | Provincial Level | Others | Che Hong County | 2015 |
| Guizhou-49 | Miao ethnic martial arts Opera | Provincial Level | Others | Pu An County | 2015 |
| Guizhou-50 | Jiadeng Opera | Provincial Level | Dengxi Opera | Weng An County | 2015 |
| Guizhou-51 | Huadengxi Opera | Provincial Level | Dengxi Opera | Xi Feng County | 2015 |
| Guizhou-52 | Huadengxi Opera | Provincial Level | Dengxi Opera | Yanhe Autonomous County along the river | 2015 |
| Guizhou-53 | Yangxi Opera | Provincial Level | Yangxi Opera | Zheng'an County | 2015 |
| Guizhou-54 | Nuo Opera | Provincial Level | Nuo Opera | Meitan County | 2015 |
| Guizhou-55 | Nuo Opera | Provincial Level | Nuo Opera | Shiqian County | 2015 |
| Guizhou-56 | Nuo Opera | Provincial Level | Nuo Opera | Nayong County | 2015 |
| Guizhou-57 | Di Opera | Provincial Level | Di Opera | Guanling Buyi and Miao Autonomous County | 2015 |
| Guizhou-58 | Wenchang Opera | Provincial Level | Others | Tongzi County | 2019 |
| Guizhou-59 | Zitong Opera | Provincial Level | Others | Daozhen Yi and Miao Autonomous County | 2019 |
| Guizhou-60 | Zitong Opera | Provincial Level | Others | Anlong County | 2019 |
| Guizhou-61 | Mulian Opera | Provincial Level | Others | Daozhen Yi and Miao Autonomous County | 2019 |
| Guizhou-62 | Dragon and Lion Festival Lanterns | Provincial Level | Others | Shibing County | 2019 |
| Guizhou-63 | Peking Opera | Provincial Level | Others | Guiyang City | 2019 |
| Guizhou-64 | Tea Lamp (High-pitched Tea Lamp) | Provincial Level | Dengxi Opera | Fenggang County | 2019 |
| Guizhou-65 | Mamadeng Opera | Provincial Level | Dengxi Opera | Suixian County | 2019 |
| Guizhou-66 | Yangxi Opera | Provincial Level | Yangxi Opera | Suixian County | 2019 |
| Guizhou-67 | Yangxi Opera | Provincial Level | Yangxi Opera | Biqiang District | 2019 |
| Guizhou-68 | Huadengxi Opera | Provincial Level | Dengxi Opera | Cen Gong County | 2019 |
| Guizhou-69 | Huadengxi Opera | Provincial Level | Dengxi Opera | Shibing County | 2019 |
| Guizhou-70 | Huadengxi Opera | Provincial Level | Dengxi Opera | Wudang District | 2019 |
| Guizhou-71 | Huadengxi Opera | Provincial Level | Dengxi Opera | Dejiang County | 2019 |
| Guizhou-72 | Pingbao Huadengxi Opera | Provincial Level | Dengxi Opera | Guanshanhu District | 2019 |
| Guizhou-73 | Liuzhi Huadengxi Opera | Provincial Level | Dengxi Opera | Lixi Special District | 2019 |
| Guizhou-74 | Buli Ethnic Group's Huadengxi Opera | Provincial Level | Dengxi Opera | Zhenfeng County | 2019 |
| Guizhou-75 | Longli Taiping Huadengxi Opera | Provincial Level | Dengxi Opera | Longli County | 2019 |
| Guizhou-76 | Dong Opera | Provincial Level | Others | Tianzhu County | 2019 |
| Guizhou-77 | Dong Opera | Provincial Level | Others | Jianhe County | 2019 |
| Guizhou-78 | Di Opera(Huaxi Dazhai Di Opera） | Provincial Level | Di Opera | Huaxi District | 2019 |
| Guizhou-79 | Gaotai Di Opera | Provincial Level | Di Opera | Puding County | 2019 |
| Guizhou-80 | Longchang Di Opera | Provincial Level | Di Opera | Ziyun Miao and Buyi Autonomous County | 2019 |
| Guizhou-81 | Zhexi Di Opera | Provincial Level | Di Opera | Lixi Special District | 2019 |
| Guizhou-82 | Zhexi Di Opera | Provincial Level | Di Opera | Gui'an New Area | 2019 |
| Guizhou-83 | Puppet Show(Guiyang Puppet Show) | Provincial Level | Puppet Show | Guiyang City | 2019 |
| Guizhou-84 | Nuo Opera | Provincial Level | Nuo Opera | Xishuang County | 2019 |
| Guizhou-85 | Nuo Opera | Provincial Level | Nuo Opera | Shuicheng County | 2019 |
| Guizhou-86 | Nuo Opera | Provincial Level | Nuo Opera | Yanhe Tujia Autonomous County | 2019 |
| Guizhou-87 | Nuo Opera | Provincial Level | Nuo Opera | Sinan County | 2019 |
| Guizhou-88 | Nuo Opera | Provincial Level | Nuo Opera | Chehen County | 2019 |
| Guizhou-89 | Wenqing Opera | Provincial Level | Others | Zhiangin County | 2019 |
| Guizhou-90 | Dengjia Opera | Provincial Level | Dengxi Opera | Fuquan City | 2019 |
| Guizhou-91 | Yao Bai Opera | Provincial Level | Others | Qinping County of Qiandongnan Miao and Dong Autonomous Prefecture | 2024 |
| Guizhou-92 | The Yi Ethnic Group's Tuotaiji | Provincial Level | Others | Hezhang County of Bijie City | 2024 |
| Guizhou-93 | Huadengxi Opera | Provincial Level | Dengxi Opera | Shuicheng County of Liupanshui City | 2024 |
| Guizhou-94 | Huadengxi Opera | Provincial Level | Dengxi Opera | Suixian County of Zunyi City | 2024 |
| Guizhou-95 | Di Opera | Provincial Level | Di Opera | Qingzhen City of Guiyang City | 2024 |
| Guizhou-96 | Nuo Opera | Provincial Level | Nuo Opera | Songtao Miao Autonomous County, Tongren City | 2024 |
| Guizhou-97 | Nuo Opera | Provincial Level | Nuo Opera | Huangping County, Qiandongnan Miao and Dong Autonomous Prefecture | 2024 |
| Guizhou-98 | Yangxi Opera | Provincial Level | Yangxi Opera | Chehen County, Qianxinan Buyi and Miao Autonomous Prefecture | 2024 |
| Yunan-1 | Huadengxi Opera（Yuxi Huadengxi Opera） | National Level | Dengxi Opera | Yuxi City, Yunnan Province | 2006 |
| Yunan-2 | Huadengxi Opera | National Level | Dengxi Opera | Yunnan Lantern Opera Troupe | 2008 |
| Yunan-3 | Huadengxi Opera | National Level | Dengxi Opera | Midu County, Yunnan Province | 2008 |
| Yunan-4 | Huadengxi Opera | National Level | Dengxi Opera | Yao'an County, Yunnan Province | 2008 |
| Yunan-5 | Huadengxi Opera | National Level | Dengxi Opera | Yuanmou County, Yunnan Province | 2008 |
| Yunan-6 | Zhuang Opera | National Level | Others | Wenshan Zhuang and Miao Autonomous Prefecture, Yunnan Province | 2008 |
| Yunan-7 | Dai Opera | National Level | Others | Dehong Dai and Jingpo Autonomous Prefecture, Yunnan Province | 2006 |
| Yunan-8 | Shadow Puppetry（Tengchong Shadow Puppetry） | National Level | Shadow Puppetry | Tengchong County, Yunnan Province | 2011 |
| Yunan-9 | Dian Opera | National Level | Others | Dian Opera Theatre of Yunnan Province | 2008 |
| Yunan-10 | Dian Opera | National Level | Others | Dian Opera Troupe of Yuxi City, Yunnan Province | 2008 |
| Yunan-11 | Dian Opera | National Level | Others | Kunming City, Yunnan Province | 2008 |
| Yunan-12 | Wa Ethnic Group's Qing Opera | National Level | Others | Tengchong County, Yunnan Province | 2008 |
| Yunan-13 | Yi Opera | National Level | Others | Damao County, Yunnan Province | 2008 |
| Yunan-14 | Bai Opera | National Level | Others | Dali Bai Autonomous Prefecture, Yunnan Province | 2008 |
| Yunan-15 | Guansuo Opera | National Level | Others | Chengjiang County, Yunnan Province | 2011 |
| Yunan-16 | Duangong Opera（Zhaotong Duangong Opera） | National Level | Duangong Opera | Zhaotong City, Yunnan Province | 2021 |
| Yunan-17 | Bai Ethnic Ggroup's Chuichuiqiang Opera | National Level | Others | Yunlong County, Dali Bai Autonomous Prefecture, Yunnan Province | 2021 |
| Yunan-18 | Nuo Opera（Guansuo Opera of Xiaotun Village） | Provincial Level | Nuo Opera | Xiaotun Village, Yangzong Town, Chengjiang County, Yuxi City | 2006 |
| Yunan-19 | Duangong Opera | Provincial Level | Duangong Opera | Zhenxiong County, Zhaotong City | 2006 |
| Yunan-20 | Duangong Opera | Provincial Level | Duangong Opera | Yiying County, Zhaotong City | 2006 |
| Yunan-21 | Duangong Opera | Provincial Level | Duangong Opera | Daguan County, Zhaotong City | 2006 |
| Yunan-22 | Duangong Opera | Provincial Level | Duangong Opera | Yanjin County, Zhaotong City | 2006 |
| Yunan-23 | Duangong Opera | Provincial Level | Duangong Opera | Qiaozhai County, Zhaotong City | 2006 |
| Yunan-24 | Duangong Opera | Provincial Level | Duangong Opera | Yongshan County, Zhaotong City | 2006 |
| Yunan-25 | Duangong Opera | Provincial Level | Duangong Opera | Weixin County, Zhaotong City | 2006 |
| Yunan-26 | Duangong Opera | Provincial Level | Duangong Opera | Suijiang County, Zhaotong City | 2006 |
| Yunan-27 | Duangong Opera | Provincial Level | Duangong Opera | Zhaoyang County, Zhaotong City | 2006 |
| Yunan-28 | Huadengxi Opera（Midu Huadengxi Opera） | Provincial Level | Dengxi Opera | Dali Bai Autonomous Prefecture, Midu County | 2006 |
| Yunan-29 | Huadengxi Opera（Chongming Huadengxi Opera） | Provincial Level | Dengxi Opera | Kunming City Songming County | 2006 |
| Yunan-30 | Huadengxi Opera（Yuanmou Huadengxi Opera） | Provincial Level | Dengxi Opera | Yuanmu County | 2006 |
| Yunan-31 | Huadengxi Opera（Yaoan Huadengxi Opera） | Provincial Level | Dengxi Opera | Yao'an County, Chuxiong Yi Autonomous Prefecture | 2006 |
| Yunan-32 | Huadengxi Opera（Jianshui Huadengxi Opera） | Provincial Level | Dengxi Opera | Jianshui County, Jianke Township, Baiyun Village, Honghe Hani and Yi Autonomous Prefecture | 2006 |
| Yunan-33 | Dian Opera | Provincial Level | Others | Yi County, Kunming City | 2006 |
| Yunan-34 | Yi Opera | Provincial Level | Others | Yongren County, Chuxiong Yi Autonomous Prefecture | 2006 |
| Yunan-35 | Bai Opera | Provincial Level | Others | Dali Bai Autonomous prefecture | 2006 |
| Tibet-1 | Tibetan Opera（Jiumulong of Lhasa） | National Level | Tibetan Opera | Tibet Autonomous Region | 2006 |
| Tibet-2 | Tibetan Opera（Jiangba of Xigaze） | National Level | Tibetan Opera | Tibet Autonomous Region | 2006 |
| Tibet-3 | Tibetan Opera（Nanmulinxiangba of Xigaze） | National Level | Tibetan Opera | Tibet Autonomous Region | 2006 |
| Tibet-4 | Tibetan Opera（Rinbungjianggar of Xigaze） | National Level | Tibetan Opera | Tibet Autonomous Region | 2006 |
| Tibet-5 | Tibetan Opera（Yalongzaxixueba of Shannan ） | National Level | Tibetan Opera | Tibet Autonomous Region | 2006 |
| Tibet-6 | Tibetan Opera（Jokhang Kachodzaxibinton of Shannan） | National Level | Tibetan Opera | Tibet Autonomous Region | 2006 |
| Tibet-7 | Tibetan Opera（Nimutarong Tibetan Opera） | National Level | Tibetan Opera | Nimu County, Tibet Autonomous Region | 2011 |
| Tibet-8 | Menba Opera of Shannan | National Level | Others | Tibet Autonomous Region | 2006 |
| Tibet-9 | Bagon (Xialbagon) | National Level | Others | Shigatse, Tibet Autonomous Region | 2021 |
| Tibet-10 | [Jiumulong of Lhasa](https://zhida.zhihu.com/search?content_id=243502126&content_type=Article&match_order=1&q=%E6%8B%89%E8%90%A8%E8%A7%89%E6%9C%A8%E9%9A%86%E8%97%8F%E6%88%8F&zd_token=eyJhbGciOiJIUzI1NiIsInR5cCI6IkpXVCJ9.eyJpc3MiOiJ6aGlkYV9zZXJ2ZXIiLCJleHAiOjE3NzU0Nzk5MTUsInEiOiLmi4nokKjop4nmnKjpmobol4_miI8iLCJ6aGlkYV9zb3VyY2UiOiJlbnRpdHkiLCJjb250ZW50X2lkIjoyNDM1MDIxMjYsImNvbnRlbnRfdHlwZSI6IkFydGljbGUiLCJtYXRjaF9vcmRlciI6MSwiemRfdG9rZW4iOm51bGx9.FEa2ERFJE6oKHnMP_QbFudv9AsJZENqqnY_bjSiAw1g&zhida_source=entity) | Provincial Level | Tibetan Opera | Lhasa Municipal Bureau of Culture | 2007 |
| Tibet-11 | Ngamthang County, Ngari Prefecture, Shigatse City (Tibetan Opera) | Provincial Level | Tibetan Opera | Qikasar City Culture Bureau | 2007 |
| Tibet-12 | Nyingba in Namuling, Shigatse (Tibetan Opera) | Provincial Level | Tibetan Opera | Qikasar City Culture Bureau | 2007 |
| Tibet-13 | Shannan Menba Agilam | Provincial Level | Others | Shannan City Culture Bureau | 2007 |
| Tibet-14 | Nepchuangjianggala, Shigatse (Tibetan Opera） | Provincial Level | Tibetan Opera | Qikasar City Culture Bureau | 2007 |
| Tibet-15 | Nyalam Chokladzhasibinoton （Tibetan Opera） | Provincial Level | Tibetan Opera | Shannan City Culture Bureau | 2007 |
| Tibet-16 | Nyalam Naidongyalongzhaxixueba (Tibetan Opera) | Provincial Level | Tibetan Opera | Shannan City Culture Bureau | 2007 |
| Tibet-17 | Qu Kuzi Ba (Tibetan Opera) | Provincial Level | Tibetan Opera | Wangkaizi County Cultural Bureau, Shannan City | 2009 |
| Tibet-18 | Rugulam | Provincial Level | Others | Luoza County Cultural Bureau, Shannan City | 2009 |
| Tibet-19 | Nimutarong (Tibetan Opera) | Provincial Level | Tibetan Opera | Nimu County Cultural Bureau of Lhasa City | 2009 |
| Tibet-20 | Chaya Xiangchi (Tibetan Opera) | Provincial Level | Tibetan Opera | Chadu Prefecture Cultural Bureau | 2009 |
| Tibet-21 | Tibetan Opera | Provincial Level | Tibetan Opera | Marbuqia Township, Sakya County, Shigatse City | 2013 |
| Tibet-22 | Jiang Da Gangtu (Tibetan Opera) | Provincial Level | Tibetan Opera | Chamdo City Jiangda County Bureau of Culture, Press, Publication and Radio and Television | 2018 |
| Tibet-23 | Gesar Armor Hymn | Provincial Level | Others | Chayi County Cultural Bureau, Chamdo City | 2024 |
| Tibet-24 | Desert Geesar Opera | Provincial Level | Others | The Cultural and Tourism Bureau of Bangong County, Naqu City | 2024 |
| Tibet-25 | Koga (Tibetan Opera) | Provincial Level | Tibetan Opera | The Cultural and Tourism Bureau of Pu'er County, Ali Region | 2024 |
| Tibet-26 | Chentang Mani Opera | Provincial Level | Others | Dingjia County Culture and Tourism Bureau of Shigatse City | 2024 |

Note: The data was collected from the official websites of China Intangible Cultural Heritage Network ([https://www.ihchina.cn/project#target1](https://www.ihchina.cn/project#target1)) and ICH list in provincial government websites.
